# Candida albicans Pacemaker Lead Endocarditis: A Case Report

**DOI:** 10.7759/cureus.79824

**Published:** 2025-02-28

**Authors:** Siham Karrati, Ibtissam Mhirig, Mahjouba Baiya, Awatif El Hakkouni

**Affiliations:** 1 Biology Department/Parasitology-Mycology Laboratory, Mohammed VI University Hospital/Faculty of Medicine and Pharmacy-Cadi Ayyad University, Marrakech, MAR

**Keywords:** antifungals, candida albicans, fungal endocarditis, pacemaker, surgical explantation

## Abstract

Fungal endocarditis (FE) is a rare yet potentially life-threatening complication of permanent cardiac pacing, most commonly caused by species of the genera *Candida* and *Aspergillus*. The diagnosis is challenging, as the clinical presentation of FE is usually nonspecific and insidious. A high level of clinical suspicion is required, particularly in patients with implantable pacemakers presenting with unexplained fever. Early diagnosis is crucial for effective and prompt management, which generally relies on a combined medical and surgical approach. We report a case of fungal pacemaker lead endocarditis caused by Candida albicans in a 68-year-old man, which was successfully treated with a multidisciplinary approach combining surgical explantation and antifungal therapy.

## Introduction

Infective endocarditis (IE) is a severe infection involving the native or prosthetic heart valves, mural endocardium, or indwelling cardiac devices [1]. It is associated with significant morbidity and mortality, particularly if not treated promptly [1,2]. Bacteria are the primary pathogens responsible for IE, with staphylococci and streptococci accounting for approximately 80% of cases [2]. However, fungal organisms can also be causative agents of IE [1,2].

Fungal endocarditis (FE) is an uncommon form of IE, representing 2% to 4% of all endocarditis cases [3], and is associated with high morbidity and mortality rates (>70%) [4]. The two most common fungi causing endocarditis are species of the genera *Candida*, implicated in 50% to 80% of cases, followed by Aspergillus [4,5]. Among *Candida *species, *Candida albicans* (*C. albicans*) remains the leading pathogen in FE, accounting for 30% to 40% of cases [3,5]. Risk factors commonly associated with FE include structural heart diseases, prosthetic heart valves, cardiac implantable electronic devices such as pacemakers (PMs) and implantable cardioverter-defibrillators (ICDs), prior heart surgery, intravenous drug use, prolonged use of broad‑spectrum antibiotics, catheter-related infections, diabetes mellitus, and immunocompromised states [3,6,7].

Pacemaker lead endocarditis commonly results from direct contamination during implantation or hematogenous spread from distant infection sites, leading to pathogen adhesion to the pacemaker leads and subsequent biofilm formation, which impairs the penetration of antimicrobial agents [4,8].

FE presents significant diagnostic and therapeutic challenges [6]. It often manifests as a form of subacute endocarditis with prolonged fever of unknown origin, which may be isolated or accompanied by other nonspecific systemic signs [3,7]. Echocardiography and blood cultures are essential diagnostic tools for FE [4]. The mainstay of FE treatment is prompt surgical intervention, coupled with optimal antifungal therapy, to reduce its exceptionally high mortality and morbidity [3,4,7].

Here, we report a case of fungal pacemaker lead endocarditis due to *C. albicans*, in which a combination of antifungal therapy and surgical explantation resulted in a successful outcome.

## Case presentation

A 68-year-old man with a medical history of type 2 diabetes mellitus, arterial hypertension, and a dual-chamber pacemaker implanted three years prior for syncopal episodes due to trifascicular block presented to the emergency department with a one-week history of fever and generalized weakness. However, the patient did not report any respiratory, urinary, or other symptoms suggestive of potential infectious foci.

On admission, the patient was conscious and febrile at 39°C, with a blood pressure of 130/70 mmHg, a pulse rate of 90 beats per minute, a respiratory rate of 17 breaths per minute, and an oxygen saturation of 96%. A complete and meticulous physical examination was performed. Upon cardiovascular examination, there was no inflammation at the pacemaker site, and cardiac auscultation revealed a grade 5/6 systolic murmur at the tricuspid focus. Other clinical examinations were unremarkable.

Suspecting infective endocarditis, multiple blood cultures were drawn, and the patient was started empirically on injectable ceftriaxone (2 g/day) and gentamicin (3 mg/kg/day) due to the frequent bacterial etiology of IE and the close similarity between bacterial and fungal causes.

Laboratory investigations revealed leukocytosis of 16,580 cells/mm³ with 70% neutrophils. Inflammatory markers were elevated, with an erythrocyte sedimentation rate (ESR) of 82 mm/h and a C-reactive protein (CRP) level of 108 mg/L. His fasting blood glucose was 181 mg/dL, and his glycated hemoglobin (HbA1c) was 8.5%. The results of other tests were normal (Table 1).

**Table 1:** Results of laboratory tests on admission.

Test	Results	Reference range
White blood cell count	16,580	4000-10,000 cells/mm³
Neutrophil count	11,606	2500-7500 cells/mm³
Hemoglobin	13.4	12-14 g/dL
Platelets	300,000	150,000-400,000 cells/mm³
Erythrocyte sedimentation rate	82	< 20 mm/h
C-reactive protein	108	< 6 mg/L
Fasting blood glucose	181	70-110 mg/dL
Glycated hemoglobin	8.5	4-6%
Blood urea nitrogen	38	25-48 mg/dL
Creatinine	1.1	0.7-1.2 mg/dL

**Figure 1:**
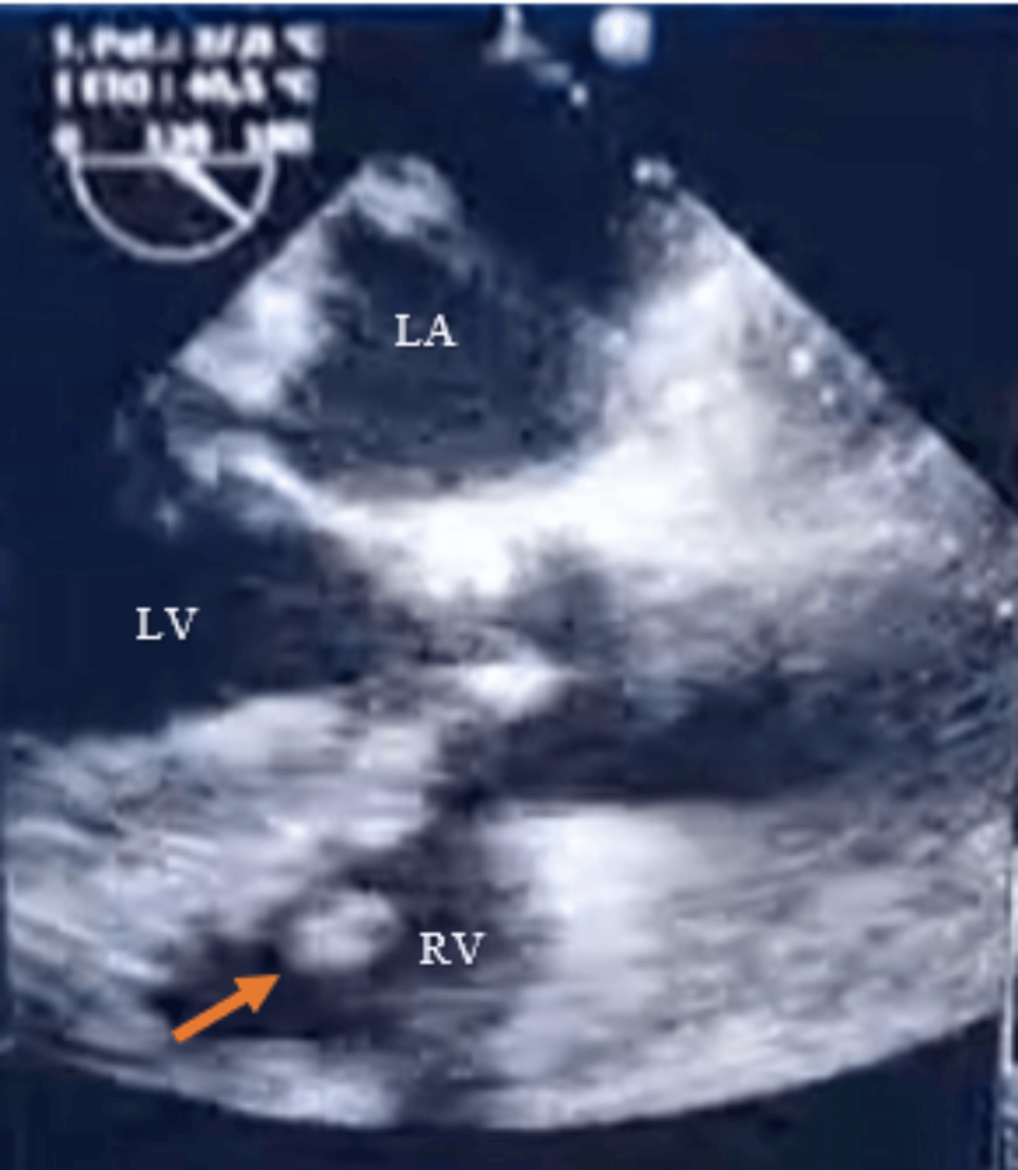
Transesophageal echocardiogram showing a large vegetation (arrow) measuring 28 × 13 mm, attached to the pacemaker lead in the right ventricle. LV: left ventricle, RV: right ventricle, LA: left atrium.

The chest X-ray was unremarkable. Transthoracic echocardiography was performed, revealing a vibratory image on the pacemaker's ventricular lead, highly suggestive of vegetation. This was later confirmed by transesophageal echocardiography, which demonstrated a vegetation attached to the ventricular lead, measuring 28 × 13 mm (Figure 1).

**Figure 2:**
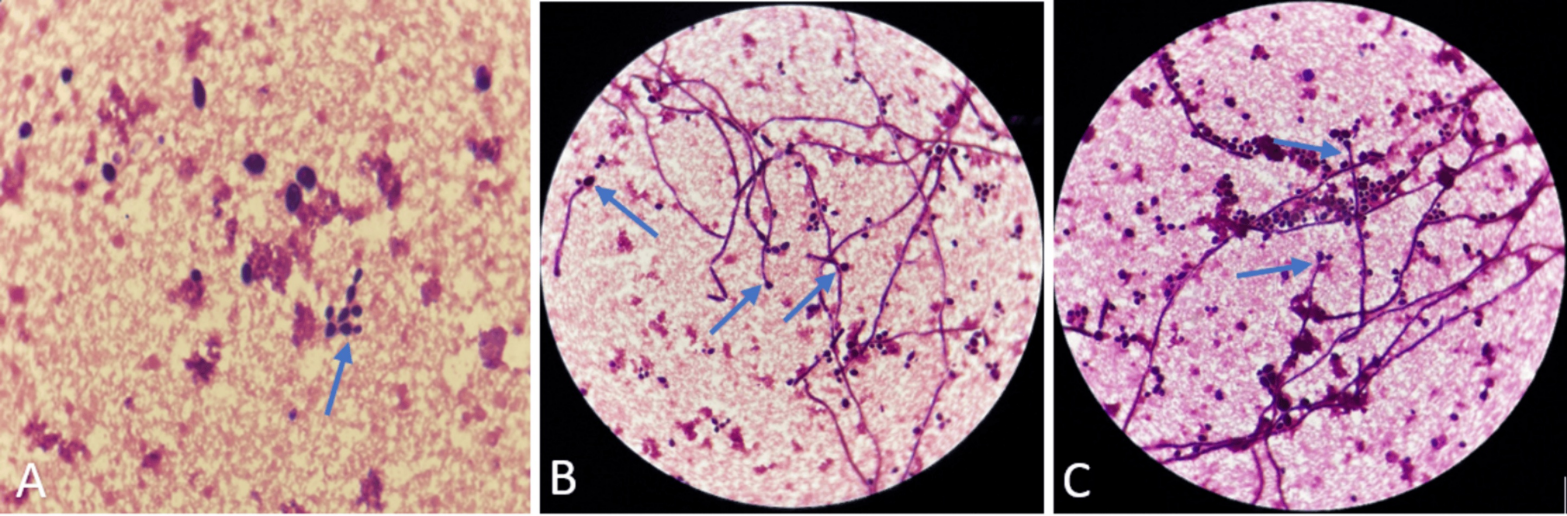
Gram-stained blood culture smears with arrows indicating budding (A) and filamentous (B, C) yeast cells (1000× magnification).

**Figure 3:**
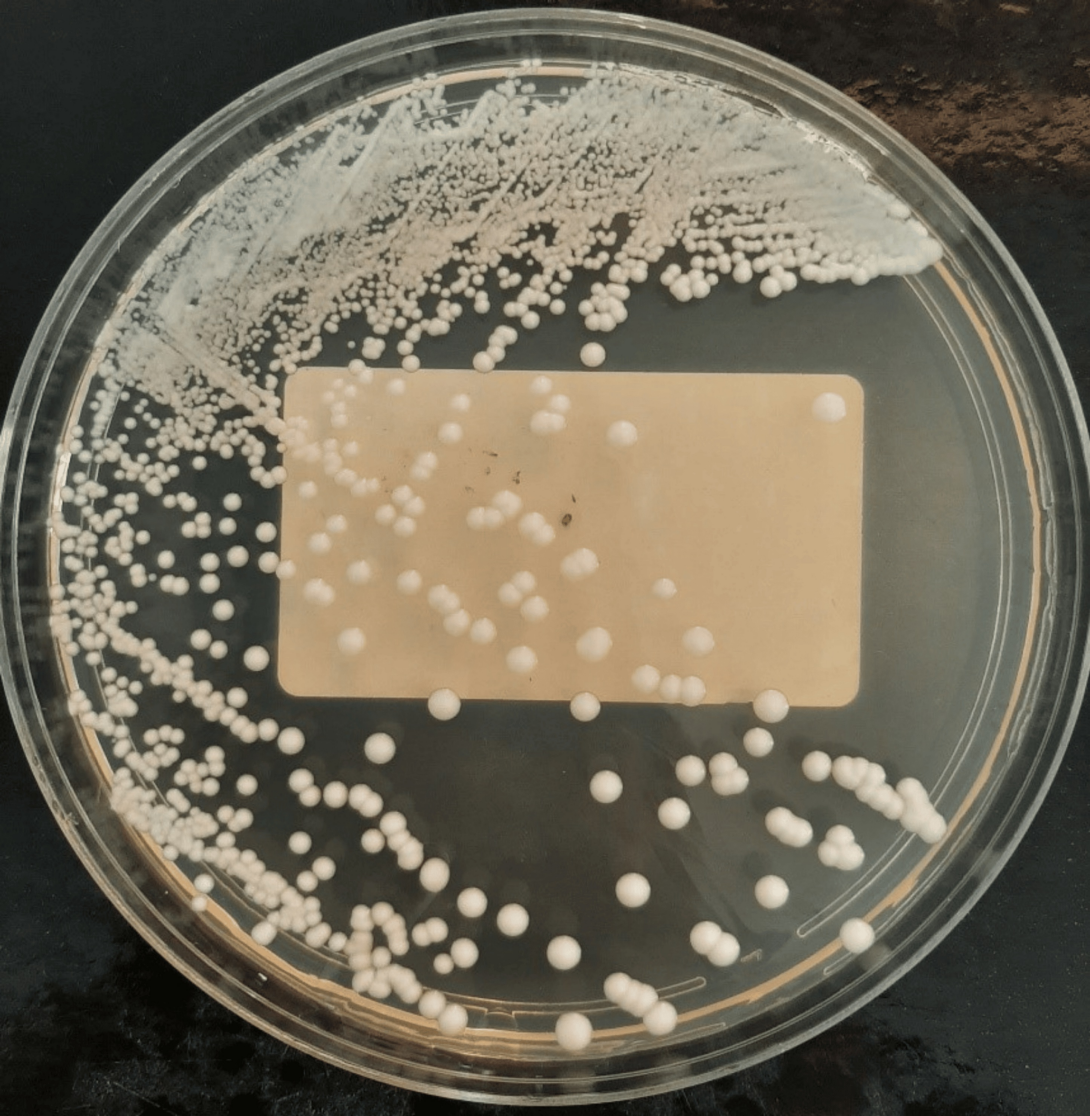
Growth of white, smooth, brilliant, and round colonies of Candida albicans on Sabouraud-chloramphenicol agar.

Two blood cultures were positive after 48 hours of incubation. Direct examination after Gram staining revealed budding and filamentous yeast cells (Figure 2). The patient was immediately started on intravenous liposomal amphotericin B, pending the results of antifungal susceptibility testing. Culture on Sabouraud-chloramphenicol agar showed the growth of numerous white, smooth, brilliant, and round colonies after 18 hours of incubation at 35°C (Figure 3). The isolated colonies were processed using VITEK 2 compact with a YST ID card (bioMérieux), which identified the yeast as C. albicans with a confidence value of 98%. The strain was sensitive to fluconazole and amphotericin B, with a minimum inhibitory concentration (MIC) ≤ 0.5 μg/mL for each; to voriconazole and caspofungin, with MIC ≤ 0.12 μg/mL for each; to micafungin, with MIC ≤ 0.06 μg/mL; and to flucytosine, with MIC ≤ 1 μg/mL.

Along with antifungal therapy, our patient underwent total removal of the pacemaker. Cultures of the vegetation and pacemaker lead were both positive for *C. albicans*.

Due to the combined treatment, the patient's clinical status improved; he became afebrile and was in good general condition. Serial blood cultures were sterile after the 12th day of hospitalization, with normal inflammatory markers.

After six weeks of treatment, the patient remained asymptomatic, with no evidence of relapse or complications after four months of follow-up, confirming the success of the treatment and his full recovery.

## Discussion

The increasing use of cardiac implantable electronic devices (CIEDs) has led to a rise in the incidence of pacemaker and implantable cardioverter-defibrillator infections [8,9]. Device-related endocarditis is relatively uncommon, accounting for approximately 10% of all device-related infections, and is an extremely lethal complication [10]. *Staphylococcus *spp. is the most frequently identified causative microorganism, responsible for 60% to 80% of CIED-associated endocarditis [9,11], whereas fungal pathogens are rare. Among the fungal causes of endocarditis, *C. albicans* is the most frequent [3,4,7].

Although *C. albicans* is generally a harmless commensal microorganism in the human microbiota, predominantly found in the oral cavity, gastrointestinal tract, and genital tract, it can become a pathogenic yeast under certain conditions. A major virulence factor of *C. albicans* is its capacity to form highly structured extracellular biofilms on biotic and abiotic surfaces, especially on intracardiac devices, prosthetic heart valves, and endovascular catheters [4,12,13].

*Candida *endocarditis is rarely seen in healthy individuals and is most commonly associated with various risk factors, including immunocompromised states, intravenous drug abuse, prosthetic heart valves, CIEDs, a prior history of endocarditis and cardiac surgery, an indwelling central venous catheter, diabetes mellitus, chronic kidney disease, prolonged antibiotic therapy, and hospital-acquired infections [4,5,12]. In our case, the patient had a documented history of diabetes mellitus and implantation of a dual-chamber pacemaker three years prior.

The diagnosis of FE is challenging due to its close resemblance to bacterial IE. The clinical presentation of *Candida *endocarditis is subacute and atypical, with fever as the predominant presenting symptom, either isolated or associated with other general signs such as weight loss, fatigue, diaphoresis, and chills [3,7]. Classical features such as Osler’s nodes, Janeway lesions, and Roth spots are infrequently observed in *Candida *endocarditis [4]. However, it is highly associated with embolic complications that can affect any organ, owing to the larger and more friable vegetation compared to bacterial endocarditis [3,4,7]. In a review by Halawa et al., 15 cases of well-defined CIED-associated *Candida *endocarditis were summarized, including 12 PMs and three ICDs. Symptoms were documented in 13 patients; fever was observed in 10, and four patients developed major fungal emboli in the pulmonary artery [10]. In the present case, the patient presented with fever and generalized weakness, without any evidence of embolic events.

Echocardiography is the primary imaging modality for diagnosing IE and should be performed in all suspected cases [14]. Transesophageal echocardiography has superior sensitivity in detecting pacemaker lead vegetation compared to transthoracic echocardiography, which, despite its accessibility and rapidity, has reduced sensitivity, particularly in the presence of intracardiac devices [4,12,14]. In contrast to bacterial endocarditis, fungal endocarditis is classically characterized by large and highly mobile vegetations, which can lead to massive embolic events [4,5]. In our case, a large vegetation on the ventricular lead of the pacemaker was visible on both transthoracic and transesophageal echocardiography. Similarly, in the review by Ahmad Halawa et al., lead vegetations were present in all patients, ranging in size from 0.5 to 7 cm [10]. Additionally, a 2013 case published by Tascini et al. reported a 2 cm vegetation adherent to the pacemaker's atrial lead, visible on both imaging modalities [15].

Blood cultures remain the gold standard for diagnosing IE and are mandatory in all suspected cases. However, their sensitivity in FE is relatively low [4,5], with more than 50% of cases yielding negative results despite echocardiographic evidence of vegetations [3,7]. In contrast to Aspergillus-related endocarditis, where the diagnostic yield of blood cultures is nearly null, with only 4% of cases presenting positive results [5,7], the majority of *Candida *endocarditis cases are associated with positive blood cultures, with a sensitivity of approximately 90% [4]. According to the review of 15 cases of CIED-associated *Candida* endocarditis by Halawa et al. [10], microbiological results identified *C. albicans* in seven patients, *Candida *parapsilosis in four patients, *Candida* tropicalis in two patients, and *Candida glabrata* in two patients. Notably, one patient was infected with both *C. albicans* and *C. glabrata*, while another case involved co-infection with *C. albicans* and *Staphylococcus epidermidis*. In our case, the patient had two positive blood cultures for *C. albicans*.

Four primary classes of antifungal agents are used to treat invasive candidiasis: polyenes, triazoles, echinocandins, and flucytosine [16]. The emergence of antifungal resistance in *C. albicans* represents a significant global public health concern [17]. In a systematic review and meta-analysis of antifungal resistance in *C. albicans* isolates from clinical settings in Iran, the pooled prevalence of antifungal resistance ranged from 0% to 26%. Specifically, resistance to fluconazole was found in 20.37% of isolates, while resistance to amphotericin B was observed in 7.28%. Additionally, the pooled prevalence of resistance to echinocandins was 4.53% for caspofungin and 1.79% for anidulafungin [18]. Given the rising antifungal resistance in *C. albicans*, susceptibility-guided therapy is critical for ensuring effective treatment, minimizing treatment failures, preserving antifungal efficacy, and reducing the spread of resistance [16-18]. In our case, *C. albicans* was susceptible to all antifungal agents tested.

Regarding the treatment of FE, it generally necessitates an urgent combined surgical and medical approach, as the prognosis with medical therapy alone is typically poor, with a mortality rate of 50% to 70% for *Candida*-related endocarditis [5]. The Infectious Diseases Society of America, the European Society of Cardiology, and the American Heart Association guidelines for the management of *Candida *endocarditis recommend liposomal amphotericin B (3-5 mg/kg/day), with or without flucytosine (25 mg/kg four times daily), or high-dose echinocandin (caspofungin 150 mg daily, micafungin 150 mg daily, or anidulafungin 200 mg daily) [16,19,20]. Alternative step-down therapy with fluconazole, 400-800 mg (6-12 mg/kg) daily, can be considered for patients with fluconazole-susceptible *Candida *isolates who have demonstrated clinical stability and cleared *Candida *from the bloodstream [16]. Antifungal therapy should be administered for a minimum duration of six weeks, in addition to the prompt surgical removal of the entire infected device [16,20]. In our case, the patient underwent surgical explantation of the pacemaker and was treated with intravenous liposomal amphotericin B for six weeks as recommended. The patient exhibited favorable clinical and biological responses to treatment.

## Conclusions

FE remains the most severe form of IE, associated with excessive morbidity and mortality. Given the increasing use of CIEDs, fungal pacemaker endocarditis is becoming more frequent and can no longer be regarded as an uncommon event. It presents substantial diagnostic and therapeutic challenges. This case highlights the importance of a multidisciplinary approach involving a high degree of clinical suspicion, prompt diagnosis, and a combination of effective antifungal therapy with surgical intervention to improve clinical outcomes and reduce mortality.
